# Improving the Dynamic Mechanical Properties of XNBR Using ILs/KH550-Functionalized Multilayer Graphene

**DOI:** 10.3390/ma12172800

**Published:** 2019-08-30

**Authors:** Duoli Chen, Chaoliang Gan, Xiaoqiang Fan, Lin Zhang, Wen Li, Minhao Zhu, Xin Quan

**Affiliations:** 1 Tribology Research Institute, School of Mechanical Engineering, Southwest Jiaotong University, Chengdu 610031, China; 2 Key Laboratory of Advanced Technologies of Materials (Ministry of Education), School of Materials Science and Engineering, Southwest Jiaotong University, Chengdu 610031, China; 3 College of Mechanical and Electrical Engineering, Yangtze Normal University, Chongqing 408100, China

**Keywords:** graphene, ionic liquids, modification, damping property

## Abstract

Graphene has been considered an ideal nanoscale reinforced phase for preparing high-performance composites, but the poor compatibility and weak interfacial interaction with the matrix have limited its application. Here a highly effective and environmentally friendly method for the functionalization of graphene is proposed through an interaction between as-exfoliated graphene and (3-aminopropyl) triethoxysilane (KH550), in which 1-butylsulfonate-3-methylimidazolium bisulfate (BSO_3_HMIm)(HSO_4_) ionic-liquids-modified graphene was prepared via an electrochemical exfoliation of graphite in (BSO_3_HMIm)(HSO_4_) solution, then (BSO_3_HMIm)(HSO_4_)-modified graphene as a precursor was reacted with amine groups of KH550 for obtaining (BSO_3_HMIm)(HSO_4_)/KH550-functionalized graphene. The final products as filler into carboxylated acrylonitrile‒butadiene rubber (XNBR) improve the dynamic mechanical properties. The improvement in the dynamic mechanical properties of the nanocomposite mainly depends on high interfacial interaction and graphene’s performance characteristics, as well as a good dispersion between functionalized graphene and the XNBR matrix.

## 1. Introduction

Nowadays, damping materials have been widely applied in the mechanical vibration field. Among the numerous damping materials, polymer composites have been extensively researched for their excellent physical and chemical properties [1,2]. However, it is a challenge to improve the damping performance without sacrificing the mechanical strength of rubber composites [3]. Carbon nanomaterials can be used to increase the damping properties of a polymer due to the frictional energy dissipation during interfacial sliding. In many reports [4,5,6,7], the uniform dispersion of carbon nanomaterials in a polymer matrix can increase the interfacial interaction and thus improve the dynamic mechanical properties. Graphene has been the focus of the search for an ideal reinforcement for preparing high-performance composites. However, the poor compatibility and weak interfacial interaction between graphene and the polymer matrix are among the most important problems to address before further application. Many methods have been used to increase the dispersion in polymer matrixes, including covalent binding with free radicals [8,9,10] and dienophiles [11,12,13], grafting through the chemistry of epoxy and hydroxyl groups of GO [14,15], and π‒π interaction with organic groups [16,17,18]. So far, there are still some difficulties in real-life application of modified graphene using these methods due to complex reaction conditions, hazardous organic solvents, difficult secondary modification, and so on. Thus, it is important to develop a highly effective and environmentally friendly method to prepare functionalized graphene.

Ionic liquids (ILs) have attracted extensive attention due to their unique properties like low toxicity, nonflammability, and recyclability [19]. Liu et al. [20] reported a green method to prepare ionic liquid functionalized multilayer graphene, in which ILs could be deposited on the surface of exfoliated multilayer graphene. This method has been regarded as a promising approach because it could circumvent the defects in graphene oxide derivatives [19]. Currently, various nanostructures, including mesoporous silica [21,22], multiwall carbon nanotubes [23,24,25], and graphene oxide (GO) [14,15], have been functionalized using ILs. Furthermore, some ILs with functional groups could be used as an efficient platform for further reaction with inorganic and organic materials [26,27,28].

In this work, a new method for the preparation of functionalized multilayer graphene was proposed. (BSO_3_HMIm)(HSO_4_) ILs-modified multilayers graphene (MLG) is an efficient platform for further grafting with KH550 to improve compatibility and interfacial interactions in the XNBR matrix. (3-aminopropyl) triethoxysilane (KH550) has been used to increase compatibility in polymer matrixes [29,30,31]. To explore the application prospects of this functionalization method, the (BSO_3_HMIm)(HSO_4_)/KH550-functionalized graphene was dispersed into a rubber matrix to estimate the dynamic mechanical properties.

## 2. Experimental 

### 2.1. Materials

1-butylsulfonate-3-methylimidazolium bisulfate (BSO_3_HMIm)(HSO_4_) ILs and high-purity graphite rods were obtained from Lanzhou Institute of Chemical Physics (Lanzhou, China). Direct-current power supply was commercially obtained from HYELEC Co., Ltd. (Hangzhou, China). XNBR (1027) with an acrylonitrile mass fraction of 27% was obtained from NANCAR Co., Ltd. (Taiwan, China). 

### 2.2. Surface Functionalization of Multilayer Graphene by (BSO_3_HMIm)(HSO_4_) and Silane Grafting

(BSO_3_HMIm)(HSO_4_)/KH550-functionalized graphene was prepared according to four steps. First, two high-purity graphite rods were inserted as electrodes into the solution of water and 1-butylsulfonate-3-methylimidazolium bisulfate with a volume ratio of 10:1. Two high-purity graphite rods were placed parallel to each other and 2 cm apart in a 50-mL beaker. Direct-current power supply was explored to provide a static potential of 5 V. After 4 h, the electrolyte became black due to the exfoliation from the anode graphite surface. Secondly, the black solution with exfoliated graphene was centrifuged at a speed of 4000 rpm. The black precipitate was washed with absolute ethanol and deionized water several times. The as-obtained ILs-modified multilayer graphene was denoted as MLG1. Thirdly, KH550 was used as a grafting agent at 60 °C for 5 h and the product was isolated by centrifugation at 4000 rpm. Finally, the functionalized multilayer graphene was thoroughly washed with deionized water to remove KH550 monomers and dried in vacuum drying oven, and the as-prepared sample was denoted as MLG1-KH550. In order to estimate the reaction efficiency between (BSO_3_HMIm)(HSO_4_) and KH550, (BSO_3_HMIm)(HSO_4_) with different concentrations was added to regulate its density on MLG’s surface. MLG2-KH550 and MLG3-KH550 were obtained by the same process with mixtures of water and (BSO_3_HMIm)(HSO_4_) at 10:2 and 10:3, respectively.

### 2.3. Characterization of Functionalized Multilayer Graphene

FTIR spectra of untreated graphite, MLG1, and MLG1-KH550 samples were obtained by a Bruker VERTEX70 FTIR spectrometer (Bruker optik, Ettlingen, Germany). X-ray photoelectron spectroscopy (ESCALAB 250Xi, ThermoFisher Scientific, USA) was employed to explore the chemical state of typical elements on the functionalized multilayer graphene. The morphology of MLG-KH550 and XNBR/MLG-KH550 composites were determined by transmission electron microscopy (FEI, Hillsboro, OR, USA). The degree of defects in carbon materials was evaluated by Raman microscopy (Horiba LabRam HR800). The thermogravimetric curves of samples were investigated with a Thermal Analyzer (STA 449C, Netzsch, Germany) at a heating rate of 20 °C/min in flowing N2. Dynamic thermomechanical analysis was explored by DMA (TA Instruments, New Castle, DE, USA, stretching mode). 

### 2.4. Preparation of Functionalized Multilayer Graphene Hybrid XNBR

A twin-roller laboratory mill was employed to mix the rubber and filler. First, the MLG1-KH550 with additional content of 1.0 wt % was added to XNBR and they were mixed at room temperature until the filler had uniform distribution in the matrix. Secondly, the rubber mixture was placed in a metal mold for vulcanization. The vulcanization temperature was set at 145 °C for 10 min and the pressure dependence was under 10 MPa. Finally, the composites with different addition content (0.2, 0.5, 1.0, and 2.0 wt %) were prepared and to explore the performance characteristics. The composites with different content were denoted as XNBR/MLG1-KH550-0.2wt %, XNBR/MLG1-KH550-0.5wt %, XNBR/MLG1-KH550-1.0wt %, and XNBR/MLG1-KH550-2.0wt %, respectively.

## 3. Results and Discussion

**Figure 1:**
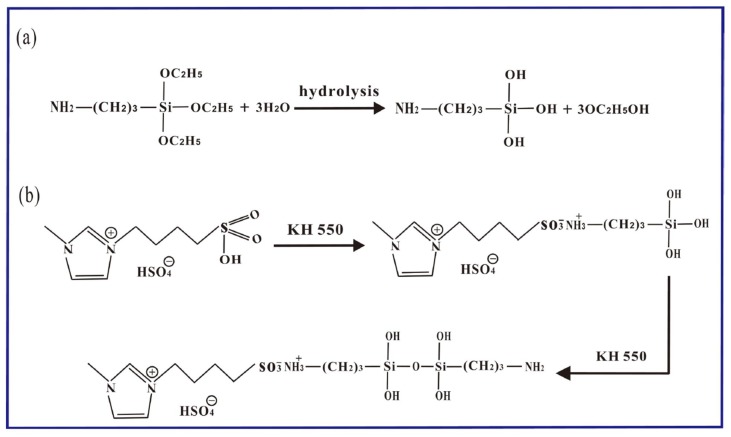
Flowchart for the fabrication process of functional multilayer graphene (MLG) and XNBR/MLG-KH550. (**a**) KH550 can easily hydrolyze in water and alkyl siloxane hydrolysis into silanol; (**b**) SO_3_H-functionalized ILs can react with amines to stabilize amino silane (KH550).

A possible reaction pathway has been proposed [32,33]. As seen in Figure 1a, KH550 can easily hydrolyze in water and alkyl siloxane hydrolysis into silanol. Then, SO_3_H-functionalized ILs can react with amines to stabilize amino silane (KH550) in Figure 1b. Finally, silanol can react directly with the hydroxyl groups. Thus, KH550 could be considered an efficient platform for secondary reactions with itself. As-prepared functionalized MLG could disperse well into the XNBR matrix to obtain a high-performance composite.

**Figure 2:**
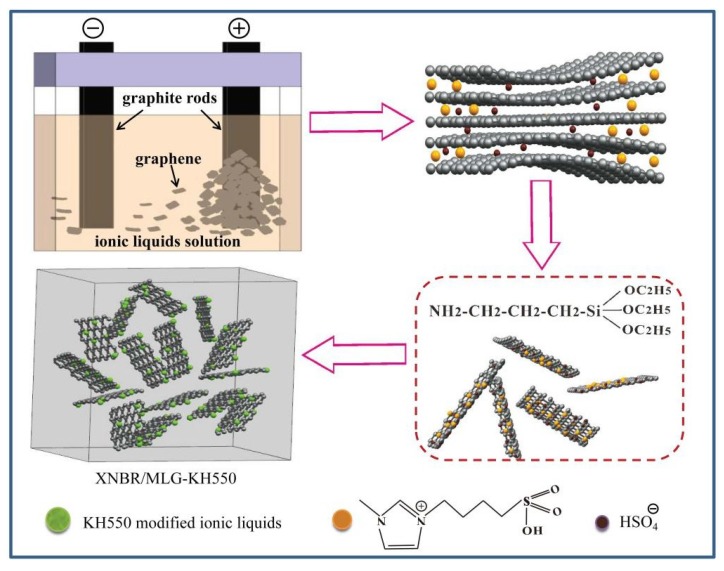
Possible chemical reactions between (BSO_3_HMIm)(HSO_4_) and KH550.

The preparation procedure of functionalized-MLG-enhanced XNBR nanocomposites is shown in Figure 2. During the fabrication process, the HSO4^–^ anions were effectively intercalated into the interlayer spacing of graphite [19]. (BSO_3_HMIm)+ cations in ILs play an important role in the formation of free radicals, like 1-octyl-3-methylimidazolium free radicals that can connect on graphene via physicochemical interaction [20] and could react with KH550.

### 3.1. Surface Functionalization of Multilayer Graphene

**Figure 3:**
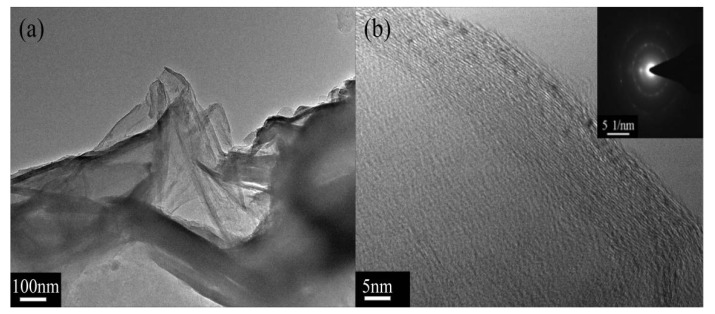

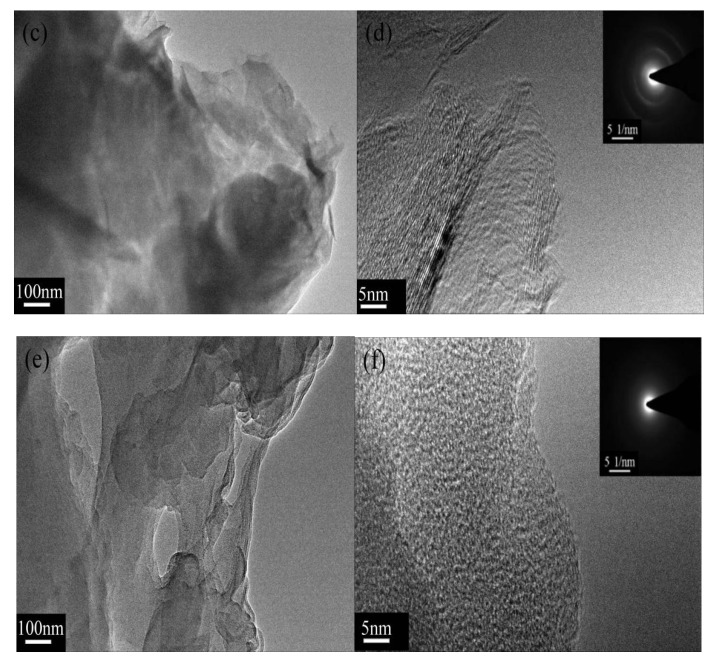
TEM images of (**a**,**b**) MLG1, (**c**,**d**) MLG2,and (**e**,**f**) MLG3.

After being modified by (BSO_3_HMIm)(HSO_4_), the surface morphology of graphene can be definitively identified by transmission electron microscopy (TEM). Figure 3 shows the structure of functional graphene created by the (BSO_3_HMIm)(HSO_4_) layer. The diffraction pattern of single-layer graphene shows a typical six-fold symmetry. Observing the TEM images of exfoliated functionalized graphene (as shown in Figure 3a,b), high-order structures are clearly visible at the edge of graphene and the amorphous structures are randomly distributed on functional graphene basal planes. More such families of spots appear with the increasing density of (BSO_3_HMIm)(HSO_4_) on the graphene surface (in Figure 3c,d). With the further increase in (BSO_3_HMIm)(HSO_4_), the amorphous structure are definitively identified and the families of spots disappear (in Figure 3e,f), indicating that a higher ILs concentration can allow graphene to obtain high-density modification.

**Figure 4:**
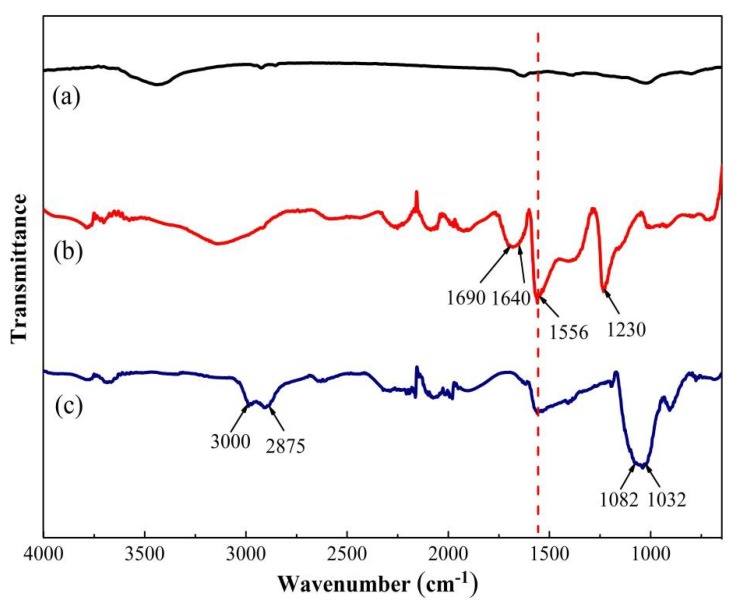
FTIR spectra of (**a**) pure graphite, (**b**) MLG1, and (**c**) MLG1-KH550.

FTIR spectra were employed to explore the chemical structure of functionalized MLG. Figure 4 shows the FTIR spectra of the untreated graphite (Figure 4a), MLG1 (Figure 4b), and MLG1-KH550 (Figure 4c), respectively. After modification with (BSO_3_HMIm)(HSO_4_) ILs, new absorption peaks at 1230 and 1566 are assigned to O–SO_2_–O stretch vibration and H–C=C–H scissoring vibration, respectively [34,35]. The new peaks at 1640 and 1690 cm^−1^ are assigned to imidazolium framework vibration, which belong to (BSO_3_HMIm)(HSO_4_) ILs. Compared with the spectra of MLG1 and MLG1-KH550, the peak at 2875 cm^−1^ is assigned to methylene stretch vibration [36]; the strong and broader characteristic peaks are located at 1032 to 1082 cm^−1^ due to Si–O–C and Si–O–Si bonds [30,33], suggesting the introduction of KH550 (in Figure 4c). The peak at 3000 cm^−1^ is assigned to the NH_3_^+^ stretching mode, indicating that ammonium groups can react with (BSO_3_HMIm)(HSO_4_) ILs [32].

**Figure 5:**
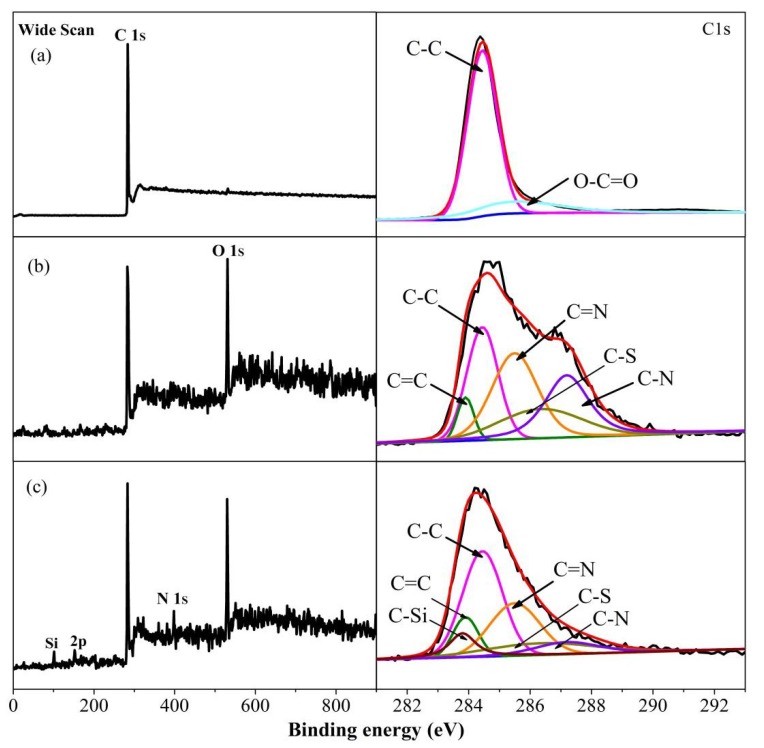
Full-range XPS spectra and high-resolution C1s spectra on (**a**) pure graphite, (**b**) MLG1, and (**c**) MLG1-KH550.

XPS was used to further analyze the chemical state of typical elements for functionalized graphene. The full-range XPS spectrum of original graphite displays only C 1s peak in Figure 5a from the graphite’s carbons, which could be fitted into C–C bonds at 284.6 eV and O–C=O bonds at 285.4 eV. For XPS of MLG1 sample in Figure 5b, the O 1s peak could be clearly distinguished from its full-range XPS spectrum; its C1s spectrum could be fitted into five different components including C–C bonds at 284.6 eV, C=C bonds at 283.9 eV, C=N bonds at 285.5 eV, C–S bonds at 286.3 eV, and C–N bonds at 287.2 eV, indicating that (BSO_3_HMIm)(HSO_4_) were successfully introduced on the interlayer/basal plane of multilayer graphene [37,38,39]. Compared with Figure 5a,b, new peaks of N1s and Si2p could be identified in the full-range spectrum of MLG1-KH550 in Figure 5c, and its C1s spectrum could be fitted into five characteristic components including C–Si (283.8 eV), C–C (284.6 eV), C=N (285.5 eV), C–N (287.2 eV), C–S (286.3 eV). A new peak at 283.8 eV represents the C–Si bonds from KH550, ensuring the successful grafting reaction between (BSO_3_HMIm)(HSO_4_) and KH550. 

The elemental ratio of MLG1-KH550, MLG2-KH550, and MLG3-KH550 was explored by XPS spectra (as shown in Table 1). The N/O ratio of MLG1-KH550 is 0.267, close to the theoretic value of 0.286 for (BSO_3_HMIm)(HSO_4_), indicating that the KH550 layer is partly covered on the graphene surface. From MLG1-KH550 to MLG3-KH550, the N/O ratio increases from 0.267 to 0.373, close to the theoretical value of 0.33 for KH550. Meanwhile, the Si/C ratio increases from 0.065 to 0.106, close to the theoretical value of 0.111 for KH550. Therefore, it can be assumed that a high concentration of ILs can effectively increase the thickness of the KH550 grafted layer.

**Figure 6:**
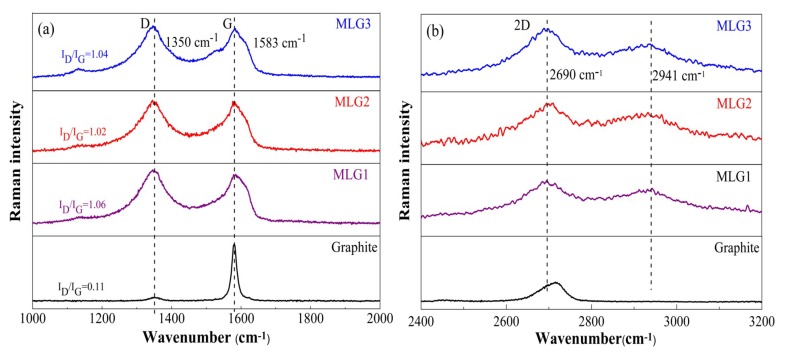
Comparison of Raman spectra for pure graphite and [BSO_3_HMIm][HSO_4_] ILs-modified multi-layers graphene (MLG). Raman spectra of (**a**) D and G band, (**b**) 2D band.

Raman spectroscopy plays an important role in the characterization of the features of sp^2^ hybridized carbon [40]. Raman spectra of graphene can provide very useful information about the change of band shift and the appearance of new peaks. Compared with pure graphite, both the characteristic peaks of D and G band become stronger and broader (Figure 6a). Meanwhile, the intensity ratio of functionalized MLG (ID/IG ≈ 1) obviously increases, indicating a successful modification [34]. In Figure 6b, a new peak at 2941 cm^−1^ further verifies that (BSO_3_HMIm)(HSO_4_) ILs are successful adhered to graphene via physicochemical interaction. Moreover, the 2D band can be used to confirm the layers of graphene, which is sensitive to graphene thickness due to the dispersion of π electrons [41]. As the layers increase, the 2D band could become broader and blue-shifted. However, the shape of the 2D band becomes hardly distinguishable between two and a few layers of graphene [42,43]. In Figure 6b, the shape of the 2D band becomes broader for the MLG, indicating that it is a multilayer structure [44]. It is worth noting that the 2D peak of functionalized MLG shifts to low frequency (red-shift), indicating that the band structure of functionalized MLG transforms into a graphene-like material (Figure 6b) [34]. 

**Figure 7:**
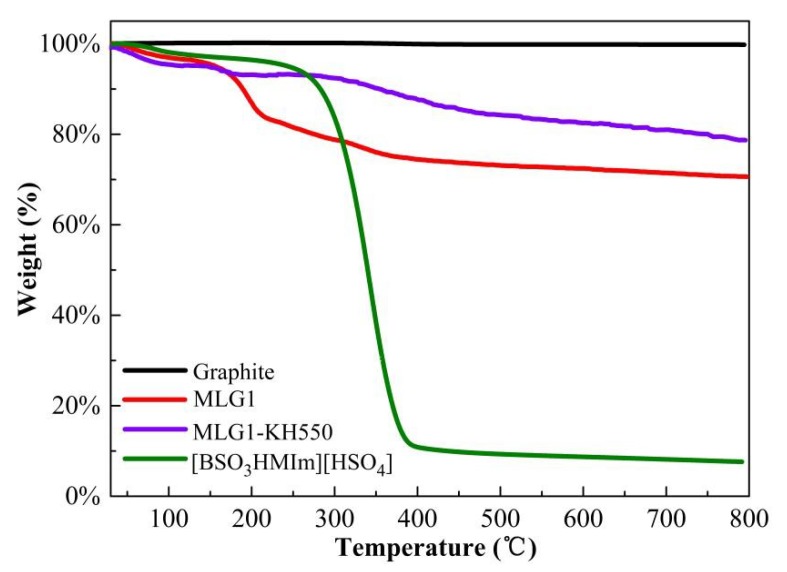
Thermogravimetric analysis results of pure graphite, MLG1, MLG1-KH550, and (BSO_3_HMIm)(HSO_4_).

Thermogravimetric (TG) curves of graphite, MLG1, MLG1-KH550, and (BSO_3_HMIm)(HSO_4_) are shown in Figure 7. The TG curve of original graphite shows almost no weight loss from 30 °C to 800 °C due to its excellent thermal stability. MLG1 causes a weight loss of 29 wt % from 30 °C to 800 °C, mainly depending on the thermal decomposition of (BSO_3_HMIm)(HSO_4_), while pure (BSO_3_HMIm)(HSO_4_) shows a serious decomposition (weight loss of approximately 98 wt %), suggesting that the higher the density of (BSO_3_HMIm)(HSO_4_) there is on graphene, the more weight loss occurs. After grafting with KH550, the weight loss of MLG1-KH550 is 19 wt %, indicating higher thermal stability of MLG1-KH550 under the same calcination procedure.

The combination of the above experimental results confirms that graphene has been functionalized by (BSO_3_HMIm)(HSO_4_) ILs and further grafting with KH550, which could offer excellent dispersion into the XNBR matrix and could enhance the interface interaction between graphene and the XNBR matrix, thereby improving the performance characteristics for practical applications.

### 3.2. Dynamic Mechanical Properties 

**Figure 8:**
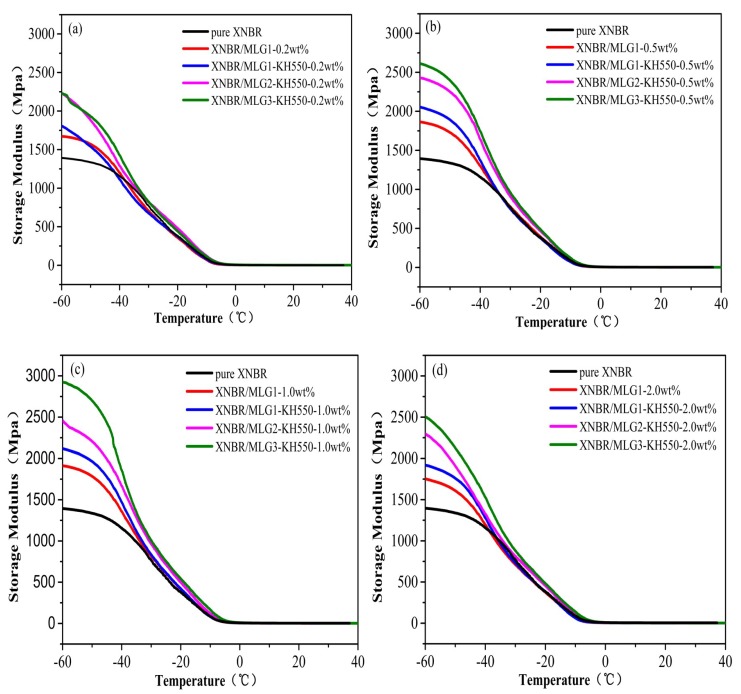
Temperature-dependent storage modulus of (**a**) XNBR/MLG-KH550-0.2wt %, (**b**) XNBR/MLG-KH550-0.5wt %, (**c**) XNBR/MLG-KH550-1.0wt %, and (**d**) XNBR/MLG-KH550-2.0wt %.

DMA is a useful tool to evaluate the interfacial interaction of polymer composites [45]. Thus we used DMA to explore the dynamic mechanical properties at a frequency of 1 Hz and a strain amplitude of 20 μm. As seen in Figure 8b–d, the storage modulus of XNBR/MLG1-KH550 is higher than the XNBR/MLG1 at a temperature range below the glass transition temperature (T_g_). From Figure 8a–d, the storage moduli of XNBR/MLG2-KH550 and XNBR/MLG3-KH550 are obviously higher than those of pure XNBR, XNBR/MLG1, and XNBR/MLG1-KH550 at a temperature range below the glass transition temperature (T_g_). As shown in Figure 8c, the storage modulus of XNBR/MLG3-KH550-1.0wt % increases to 2926 MPa at −60 °C, which gives the highest storage modulus. With the increase in filler content, the storage modulus of XNBR/MLG3-KH550-2.0wt % decreases and is close to that of XNBR/MLG2-KH550-2.0wt % in Figure 8d. It is interesting to note that KH550-grafted graphene with a higher density can effectively increase the storage modulus. This mainly depends on the high interfacial interaction and graphene’s performance characteristics between functionalized graphene and the XNBR matrix.

**Figure 9:**
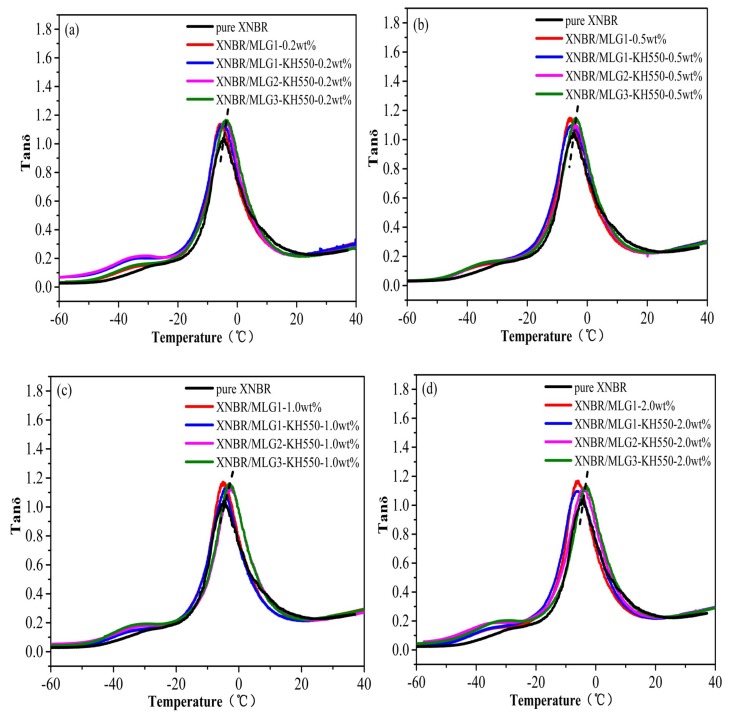
Temperature-dependent loss factors (tanδ) of (**a**) XNBR/MLG-KH550-0.2wt %, (**b**) XNBR/MLG-KH550-0.5wt %, (**c**) XNBR/MLG-KH550-1.0wt %, and (**d**) XNBR/MLG-KH550-2.0wt %.

From Figure 9a–d, the loss factors ( tanδ ) of all nanocomposite samples are higher than the pure XNBR. The T_g_ of XNBR/MLG1 and XNBR/MLG1-KH550 are obviously shifted to a lower temperature from Figure 9a–d, indicating a decrease in the cross-link density in the XNBR matrix [46]. The T_g_ of XNBR/MLG2-KH550 and XNBR/MLG3-KH550 are obviously higher than that of pure XNBR. In previous works [47,48], similar results have been reported due to the improvement of interfacial interaction between fillers and the matrix. 

### 3.3. Morphology of XNBR Composites

**Figure 10:**
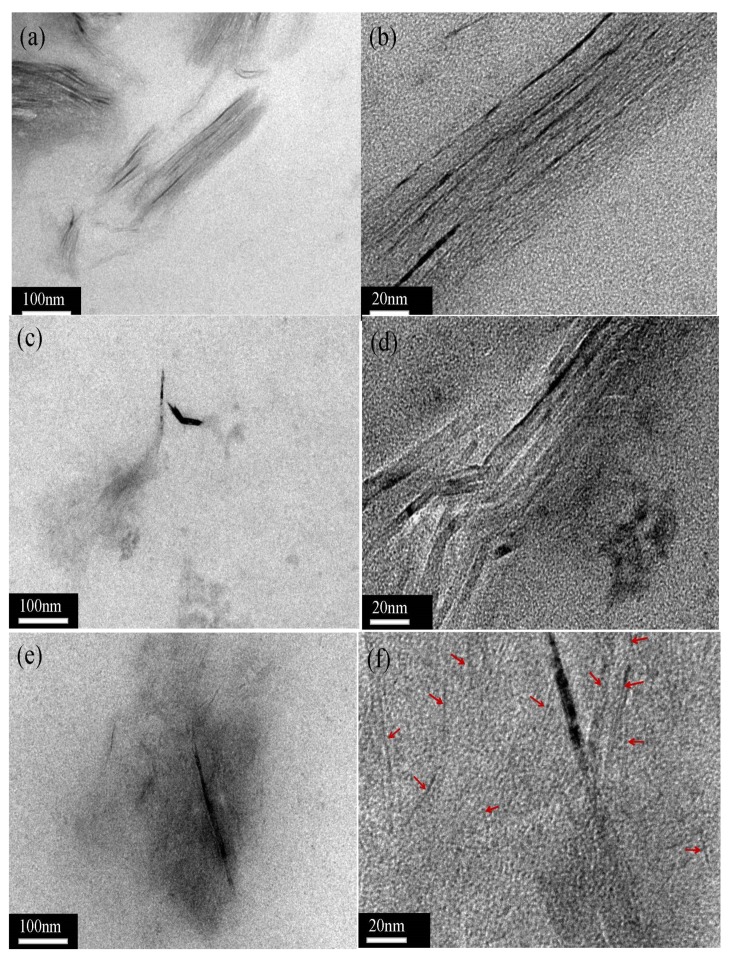
HRTEM images of (**a**,**b**) XNBR/MLG1-KH550-1wt %, (**c*,*d**) XNBR/MLG2-KH550-1wt %, and (**e**,**f**) XNBR/MLG3-KH550-1wt % composites.

The dispersity of the functionalized graphene sheets in the XNBR matrix was characterized by TEM images. The darker lines are viewed as a layer structure of functionalized graphene sheets, while the gray regions are identified as the XNBR matrix. As shown in Figure 10a,b, the MLG1-KH550 sheets with a thin grafted layer of KH550 formed an apparent stack in the XNBR matrix due to the hydrophobic interactions of graphene. Similar images have been reported in previous studies [49,50]. In Figure 10c, the stacking of MLG2-KH550 sheets causes an obvious decline in the XNBR matrix. At the higher magnification in Figure 10d, it can be observed that the graphene sheets with a thicker grafted layer of KH550 are loosely stacked. It can be seen from Figure 10e,f that MLG3-KH550 sheets with the thickest grafted layer of KH550 are well dispersed in the XNBR matrix (regions highlighted by red arrows). Therefore, it can be assumed that a higher grafted layer of KH550 can prevent the overstacking of graphene sheets in the XNBR matrix, thereby improving the corresponding dynamic mechanical properties.

To allow for practical application of high-performance rubbers, a high-efficiency and environmentally friendly method has been proposed to prepare functionalized multilayer graphene as a filler. This work demonstrates that multilayer graphene can be modified by (BSO_3_HMIm)(HSO_4_) via an electrochemical exfoliation of the graphite procedure and can be further grafted with KH550 via (BSO_3_HMIm)(HSO_4_) ILs as a high-performance platform for interactions. The efficient modification of graphene improves its compatibility in the XNBR matrix. The functionalized multilayer graphene greatly improves the dynamic mechanical properties of XNBR, which we can attribute to the enhancement in the interaction between functionalized multilayer graphene and the XNBR matrix.

## 4. Conclusions

Functionalized multilayer graphene was prepared via electrochemical exfoliation of graphite with in situ modification of ionic liquids and further grafting of KH550. As-prepared graphene as a filler in XNBR was investigated for its dynamic mechanical properties. The following conclusions can be obtained:

(a) Functionalized graphene prepared by electrochemical exfoliation of graphite in an ionic liquids solution and further grafting of KH550 is an environmentally friendly and high-efficiency method for the improvement and development of graphene-like materials;

(b) KH550-grafted graphene with a higher density can effectively enhance the dynamic mechanical properties, mainly due to the beneficial interactions of functionalized graphene and the XNBR matrix;

(c) KH550-grafted graphene as a nanoscale reinforcement can overcome stacking problems and enhance the dynamic mechanical properties. So, it is of great significance for preparing a high-performance nanocomposite for real-world applications.

## Figures and Tables

**Table 1:** Atomic ratio of MLG1-KH550, MLG2-KH550, and MLG3-KH550 measured by XPS analysis.

Samples	Element Content (at %)				Ratio of Elements	
	C	O	N	Si	N/O	Si/C
MLG1-KH550	78.54	12.89	3.45	5.12	0.267	0.065
MLG2-KH550	69.83	17.16	6.12	6.89	0.357	0.099
MLG3-KH550	68.26	17.83	6.65	7.26	0.373	0.106

## References

[1] Lv X.S., Huang Z.X., Huang C., Shi M.X., Gao G.B., Gao Q.Q. (2016). Damping properties and the morphology analysis of the polyurethane/epoxy continuous gradient IPN materials. Compos. Part. B Eng..

[2] Sasikumar K., Manoj N.R., Mukundan T., Khastgir D. (2016). Hysteretic damping in XNBR−MWNT nanocomposites at low and high compressive strains. Part. B Eng..

[3] Suhr J., Nikhil K., Pawel K., Pulickel A. (2005). Viscoelasticity in carbon nanotube composites. Nat. Mater..

[4] Chen B.Y., Ma N., Xin B., Zhang H.M., Zhang Y. (2012). Effects of graphene oxide on surface energy, mechanical, damping and thermal properties of ethylene-propylene-diene rubber/petroleum resin blends. RSC Adv..

[5] Liu S., Chen F., Zhang Y.H., Shen Q., Huang Z.X., Kemp K.C., Zhang L.M. (2014). Interfacial bond dependence of damping properties of carbon nanotubes enhanced polymers. Polym. Compos..

[6] Li B., Olson E., Perugini A., Zhong W.H. (2011). Simultaneous enhancements in damping and static dissipation capability of polyetherimide composites with organosilane surface modified graphene nanoplatelets. Polymer.

[7] Zhang C.M., Chen Y.J., Li H., Liu H.Z. (2018). Facile fabrication of polyurethane/ epoxy IPNs filled graphene aerogel with improved damping, thermal and mechanical properties. RSC Adv..

[8] Jin Z., Lomeda J.R., Price B.K., Lu W., Zhu Y., Tour J.M. (2009). Mechanically assisted exfoliation and functionalization of thermally converted graphene sheets. Chem. Mater..

[9] Sharma R., Baik J.H., Perera C.J., Strano M.S. (2010). Anomalously large reactivity of single graphene layers and edges toward electron transfer chemistries. Nano Lett..

[10] Md Z.H., Walsh M.A., Hersam M.C. (2010). Scanning tunneling microscopy, spectroscopy, and nanolithography of epitaxial graphene chemically modified with aryl moieties. J. Am. Chem. Soc..

[11] Strom T.A., Dillon E.P., Hamilton C.E., Barron A.R. (2010). Nitrene addition to exfoliated graphene: A one-step route to highly functionalized graphene. Chem. Commun..

[12] Vadukumpully S.J., Paul J., Manta N., Valiyaveettil S. (2011). Flexible conductive graphene/poly(vinyl chloride) composite thin films with high mechanical strength and thermal stability. Carbon.

[13] He H.K., Gao C. (2010). General approach to individually dispersed, highly soluble, and conductive graphene nanosheets functionalized by nitrene chemistry. Chem. Mater..

[14] Hernandez Y., Nicolosi V., Lotya M., Blighe F.M., Sun Z., De S., McGovern I.T., Holland B., Byrne M., Gun’Ko Y.K. (2008). High-yield production of graphene by liquid-phase exfoliation of graphite. Nat. Nanotechnol..

[15] Saxena A.P., Deepa M., Joshi A.G., Bhandari S., Srivastava A.K. (2011). Poly(3,4-ethylenedioxythiophene)-ionic liquid functionalized graphene/reduced graphene oxide nanostructures: Improved conduction and electrochromism. ACS Appl. Mater. Interfaces.

[16] An X.H., Butler T.W., Washington M., Nayak S.K., Kar S. (2011). Optical and sensing properties of 1-pyrenecarboxylic acid-functionalized graphene films laminated on polydimethylsiloxane membranes. ACS Nano.

[17] Xu Y.X., Bai H., Lu G.W., Li C., Shi G.Q. (2008). Flexible graphene films via the filtration of water-soluble noncovalent functionalized graphene sheets. J. Am. Chem. Soc..

[18] Cheng H.C., Shiue R.J., Tsai C.C., Wang W.H., Chen Y.T. (2011). High-quality graphene p-n junctions via resist-free fabrication and solution-based noncovalent functionalization. ACS Nano..

[19] Lu J., Yang J.X., Wang J.Z., Lim A., Wang S., Loh K.P. (2009). One-pot synthesis of fluorescent carbon nanoribbons, nanoparticles, and graphene by the exfoliation of graphite in ionic liquids. ACS Nano..

[20] Liu N., Luo F., Wu H.X., Liu Y.H., Zhang C., Chen J. (2008). One-step ionic-liquid-assisted electrochemical synthesis of ionic-liquid-functionalized graphene sheets directly from graphite. Adv. Funct. Mater..

[21] Hana P., Zhang H.M., Qiu X.P., Ji X.L., Gao L.X. (2008). Palladium within ionic liquid functionalized mesoporous silica SBA-15 and its catalytic application in room-temperature Suzuki coupling reaction. J. Mol. Catal. A-Chem..

[22] Shim H.L., Udayakumar S., Yu J.I., Kim I., Park D.W. (2009). Synthesis of cyclic carbonate from allyl glycidyl ether and carbon dioxide using ionic liquid-functionalized amorphous silica. Catal. Today.

[23] Li X.Y., Liu Y.X., Zheng L.C., Dong M.J., Xue Z.H., Lu X.Q., Liu X.H. (2013). A novel nonenzymatic hydrogen peroxide sensor based on silver nanoparticles and ionic liquid functionalized multiwalled carbon nanotube composite modified electrode. Electrochim. Acta..

[24] Wang Z.J., Zhang Q.X., Kuehner D., Xu X.Y., Ivask A., Niu L. (2008). The synthesis of ionic-liquid-functionalized multiwalled carbon nanotubes decorated with highly dispersed Au nanoparticles and their use in oxygen reduction by electrocatalysis. Carbon.

[25] Fan X.Q., Wang L.P. (2015). Ionic liquids gels with in situ modified multiwall carbon nanotubes towards high-performance lubricants. Tribol. Int..

[26] Liao H.G., Wu H., Wang J., Liu J., Jiang Y.X., Sun S.G., Lin Y.H. (2010). Direct electrochemistry and electrocatalysis of myoglobin immobilized on graphene-ctab-ionic liquid nanocomposite film. Electroanal.

[27] Qiu Y.Y., Qu X.J., Dong J., Ai S.Y., Han R.X. (2011). Electrochemical detection of DNA damage induced by acrylamide and its metabolite at the graphene-ionic liquid-Nafion modified pyrolytic graphite electrode. J. Hazard. Mater..

[28] Chai J., Li F.H., Hu Y.W., Zhang Q.X., Hana D.X., Niu L. (2011). Hollow flower-like AuPd alloy nanoparticles: one step synthesis, self-assembly on ionic liquid-functionalized graphene, and electrooxidation of formic acid. J. Mater. Chem..

[29] Gu L.L., Li T., Xu Y.J., Sun C.H., Yang Z.Y., Zhu D.L., Chen D.L. (2019). Effects of the particle size of BaTiO_3_ fillers on fabrication and dielectric properties of BaTiO_3_/Polymer/Al films for capacitor energy-storage application. Materials.

[30] Ge M.L., Wang X.B., Du M.Y., Liang G.D., Hu G.Q., Jahangir Alam S.M. (2019). Adsorption analyses of phenol from aqueous solutions using magadiite modified with organo-functional groups: Kinetic and equilibrium studies. Materials.

[31] Liu X.X., Chen X.F., Ren J.L., Zhang C.H. (2018). TiO_2_-KH550 nanoparticle- reinforced PVA/xylan composite films with multifunctional properties. Materials.

[32] Li Z.H., Yang Q.S., Qi X.D., Xu Y.Y., Zhang D.S., Wang Y.J., Zhao X.Q. (2012). A novel hydroxylamine ionic liquid salt resulting from the stabilization of NH_2_OH by a SO_3_H-functionalized ionic liquid. Chem. Commun..

[33] Wei B.G., Chang Q., Bao C.X., Dai L., Zhang G.Z., Wu F.P. (2013). Surface modification of filter medium particles with silane coupling agent KH550. Colloid. Surf. A.

[34] Fan X.Q., Wang L.P. (2015). High-performance lubricant additives based on modified graphene oxide by ionic liquids. Colloid. Interface Sci..

[35] Fan X.Q., Wang L.P., Li W. (2015). In situ fabrication of low-friction sandwich sheets through functionalized graphene crosslinked by Ionic Liquids. Tribol. Lett..

[36] Sa R., Yan Y., Wei Z.H., Zhang L.Q., Wang W.C., Tian M. (2014). Surface modification of aramid fibers by bio-Inspired poly(dopamine) and epoxy functionalized silane grafting. ACS Appl. Mater. Interfaces.

[37] Zangmeister C.D., Ma X.F., Zachariah M.R. (2012). Restructuring of graphene oxide sheets into monodisperse nanospheres. Chem. Mater..

[38] Wu J.J., Wu C.T., Liao Y.C., Lu T.R., Chen L.C., Chen K.H., Hwa L.G., Kuo C.T., Ling K.J. (1999). Deposition of silicon carbon nitride films by ion beam sputtering. Thin Solid Films..

[39] Eustatiu I.G., Tyliszczak T., Cooper G., Hitchcock A.P., Turci C.C., Rocha A.B., Barbatti M., Bielschowsky C.E. (2007). Experimental and theoretical study of S 2p and C 1s generalized oscillator strengths in CS2. J Electron. Spectrosc..

[40] Ni Z.H., Wang Y.Y., Yu T., Shen Z.X. (2008). Raman spectroscopy and imaging of graphene. Nano Res..

[41] Cancado L.G., Reina A., Kong J., Dresselhaus M.S. (2008). Geometrical approach for the study of G’ band in the Raman spectrum of monolayer graphene, bilayer graphene, and bulk graphite. Phys. Rev. B.

[42] Wang Y.Y., Ni Z.H., Yu T., Wang H.M., Wu Y.H., Chen W., Wee A.T.S., Shen Z.X. (2008). Raman studies of monolayer graphene: the substrate effect. J. Phys. Chem. C.

[43] Ferrari A.C., Meyer J.C., Scardaci V., Casiraghi C., Lazzeri M., Mauri F., Piscanec S., Jiang D., Novoselov K.S., Roth S. (2006). Raman spectrum of graphene and graphene layers. Phys. Rev. Lett..

[44] Fu H.M., Fan X.Q., Li W., Zhu M.H., Peng J.F., Li H. (2017). In situ modified multilayer graphene toward high-performance lubricating additive. RSC Adv..

[45] Xiong Y., Chen G.S., Guo S.Y. (2006). The preparation of core-shell CaCO_3_ particles and its effect on mechanical property of PVC composites. J. Appl. Polym. Sci..

[46] Luo Y., Zhao Y., Cai J., Duan Y., Du S. (2012). Effect of amino-functionalization on the interfacial adhesion of multi-walled carbon nanotubes/epoxy nanocomposites. Mater. Des..

[47] Chen S.S., Cao Y.W., Feng J.C. (2014). Polydopamine as an efficient and robust platform to functionalize carbon fiber for high-performance polymer composites. ACS Appl. Mater. Interfaces.

[48] Brocks T., Cioffi M.O.H., Voorwald H.J.C. (2013). Effect of fiber surface on flexural strength in carbon fabric reinforced epoxy composites. Appl. Surf. Sci..

[49] Ning N.Y., Ma Q., Liu S., Tian M., Zhang L.Q., Nishi T. (2015). Tailoring dielectric and actuated properties of elastomer composites by bioinspired poly(dopamine) encapsulated graphene oxide. ACS Appl. Mater. Interfaces.

[50] Lin Y., Chen Y.Z., Zeng Z.K., Zhu J.R., Wei Y., Li F.C., Liu L. (2015). Effect of ZnO nanoparticles doped graphene on static and dynamic mechanical properties of natural rubber composites. Compos. Part A appl. Sci. Manuf..

